# FGF2 Inhibits Early Pancreatic Lineage Specification during Differentiation of Human Embryonic Stem Cells

**DOI:** 10.3390/cells9091927

**Published:** 2020-08-20

**Authors:** Rabea Dettmer, Karsten Cirksena, Julia Münchhoff, Jasmin Kresse, Ulf Diekmann, Isabell Niwolik, Falk F. R. Buettner, Ortwin Naujok

**Affiliations:** Institute of Clinical Biochemistry, Hannover Medical School, Carl-Neuberg-Str.1, 30625 Hannover, Germany; dettmer.rabea@mh-hannover.de (R.D.); cirksena.karsten@mh-hannover.de (K.C.); j.muenchhoff@gmail.com (J.M.); tatzel@hotmail.de (J.K.); UlfDiekmann@gmx.de (U.D.); niwolik.isabell@mh-hannover.de (I.N.); Buettner.Falk@mh-hannover.de (F.F.R.B.)

**Keywords:** human pluripotent stem cells, pancreas organogenesis, secretomics, PDX1

## Abstract

Growth factors are important regulators during organ development. For many vertebrates (but not humans) it is known how they contribute to the formation and expansion of PDX1-positive cells during pancreas organogenesis. Here, the effects of the fibroblast growth factors FGF2, FGF7, FGF10, and epidermal growth factor (EGF) on pancreas development in humans were assessed by using human pluripotent stem cells (hPSCs). During this, FGF2 was identified as a potent anti-pancreatic factor whereas FGF7, FGF10, and EGF increased the cell mass while retaining PDX1-positivity. FGF2 increased the expression of the anti-pancreatic factor sonic hedgehog (*SHH*) while suppressing *PDX1* in a dose-dependent manner. Differentiating cells secreted SHH to the medium and we interrogated the cells’ secretome during differentiation to globally examine the composition of secreted signaling factors. Members of the TGF-beta-, Wnt-, and FGF-pathways were detected. FGF17 showed a suppressive anti-pancreatic effect comparable to FGF2. By inhibition of specific branches of FGF-receptor signaling, we allocated the *SHH*-induction by FGF2 to MEK/ERK-signaling and the anti-pancreatic effect of FGF2 to the receptor variant FGFR1c or 3c. Altogether, we report findings on the paracrine activity of differentiating hPSCs during generation of pancreatic progenitors. These observations suggest a different role for FGF2 in humans compared to animal models of pancreas organogenesis.

## 1. Introduction

Human pluripotent stem cells (hPSCs), such as embryonic stem or induced pluripotent stem cells (hESCs/hiPSCs), have emerged as a potential cell source for cell replacement therapies and powerful tools to study human development in a dish. Typically, embryonic processes are assessed by animal models of development such as mice, chick, frog, and fish. However, it remains an open question whether these models reflect the specific situation in humans. Thus, in vitro differentiation of hESCs/hiPSCs represents an alternative to assess human embryogenesis. The development of the pancreas has been extensively studied in mice. It is clear now that pancreatic organogenesis is controlled by extensive tissue interactions between the developing endoderm and its nearest neighbors the splanchnic mesoderm, the notochord, and the epithelium of the dorsal aortae (reviewed in [1,2]). A key event in this patterning process is the secretion of growth factors and signaling molecules from the mesoderm to control the first and second transition of the developing pancreas. During the first transition, epithelial PDX1-positive cells emerge from the posterior foregut endoderm and branch by massive cell proliferation into two structures, namely the ventral and the dorsal pancreatic buds. Multipotent progenitor cells (MPC) within the buds, positive for PDX1, NKX6.1, and SOX9 serve then as the cell source for further ductal, exocrine, and endocrine development. Endocrine progeny is marked by NGN3 expression in the second transition followed by the first appearance of fetal islet cells positive for insulin, glucagon, and somatostatin [1,2].

Fibroblast growth factors (FGF) such as FGF2, FGF7 (KGF), and FGF10 play a major role during the first transition period. In a pioneering study, Hebrok and colleagues identified sonic hedgehog (SHH) in chicks as a crucial anti-pancreatic signaling molecule. SHH is expressed along the endodermal epithelium of the primitive gut tube and prevented the development of the dorsal pre-pancreatic plate in an autocrine fashion. Activin-βB and FGF2 secreted by the Notochord, however, repressed SHH and permitted pancreatic lineage progression [3]. In mice, indian hedgehog (IHH) in addition to SHH regulates pancreatic fate in a comparable manner [4]. In further studies using pancreatic explants from rodents it could been shown that FGF7 and FGF10 enhance proliferation of PDX1-positive pancreatic epithelial cells and repress further differentiation towards endocrine progeny via the FGFR2IIIb receptor [5,6]. Epidermal growth factor (EGF) was also shown to enhance proliferation of undifferentiated pancreatic epithelial cells [7]. Consequently these growth factors are used after definitive endoderm (DE) induction to expand the cell population of PDX1-positive pancreatic progenitors during differentiation of hESC/hiPSC into stem cell-derived beta cells [8,9,10,11].

Recently we studied the mechanisms of anterior–posterior patterning of definitive endoderm derived from hESCs [12]. Retinoic acid-signaling and inhibition of BMP/Wnt-signaling was identified as prerequisite for the patterning of hESC-derived DE into PDX1-positive posterior foregut endoderm. These pancreatic-duodenal cells could then be further differentiated into PDX1+/NKX6.1+ multipotent progenitor cells [12,13]. In the present study we analyzed the additional effects of the growth factors FGF2, FGF7, FGF10, and EGF during the anterior–posterior patterning stage. Surprisingly, FGF2 turned out to be a very effective anti-pancreatic growth factor, potentially by inducing SHH gene expression and secretion. Potentially, differentiating PSCs might influence their cell fate by secretion of signaling molecules. In order to decipher the repertoire of secreted proteins that potentially affect the differentiation process, we performed quantitative secretomics of cell culture supernatants of hESCs and DE cells. We identified a substantial number of secreted growth factors that are potentially detrimental to pancreatic lineage progression. Amongst them, we identified FGF8 and FGF17. FGF8 and FGF17 are FGF-family members that, similar to FGF2, act by binding to the receptor splice variant FGFR1c/3c.

## 2. Materials and Methods

### 2.1. Human ESC Culture and Differentiation

Feeder-free culturing of human HES3 cells was performed as described earlier [13,14]. Differentiation was initiated according to our protocol described in [15]. Briefly, in stage 1, advanced RPMI 1640 supplemented with 50 ng/mL activin A (Peprotech, Rocky Hill, NJ, USA), 5 µM CHIR-99021 (CHIR, Cayman Chemicals, Ann Arbor, MI, USA), 1% penicillin/streptomycin, 1% Glutamax and 0.2% fetal bovine serum (FBS) were used for 24 h followed by 72 h using the same medium lacking CHIR. For differentiation into PDX1-positive pancreatic progenitor cells (stage 2), advanced RPMI 1640 plus 1–2 µM all-trans retinoic acid (ATRA), 1 µM LDN-193189 (Sigma-Aldrich, St. Louis, MO, USA) and 4 µM IWR-1 (Selleck Chemicals, Houston, TX, USA) were used [12]. Various growth factors and small molecules were supplemented to stage 2 medium: EGF or FGF2 (Reliatech, Wolfenbüttel, Germany), FGF7 or FGF10 (Peprotech), 10 µM U0126 (ERK inhibition, Selleck Chemicals), 5 µM LY294002 (PI3-kinase inhibition, Selleck Chemicals), 1 µM CP-690550 (JAK3-inhibition, Selleck Chemicals), 1 µM SP600125 (JNK inhibition, Selleck Chemicals), 10 µM SB203580 (p38 inhibition, selfmade), 10 µM Gant 58 and 2.5 µM Sant-1 (SHH-inhibition, Tocris & Selleck Chemicals) and 100 nM PD-173074 (FGFR1c/3c inhibition, Cayman Chemical Company, Ann Arbor, MI, USA). Cell numbers were assessed by flow cytometry on a CyFlow ML flow cytometer (SysmexPartec, Görlitz, Germany).

### 2.2. Gene and Expression Analysis

Total RNA was isolated using the peqGOLD RNAPure kit (Peqlab, Erlangen, Germany) following the manufacturer’s instructions. cDNA was synthesized from 1–2 µg total RNA and subsequently 5–10 ng was used for each RT-qPCR reaction. Samples were measured in triplicate using specific primer pairs on a ViiA7 system (Thermo Fisher Scientific, Waltham, MA, USA) (Supplementary Table S1). Data normalization into calibrated normalized relative quantities (CNRQ) was performed with qBasePlus (Biogazelle, Gent, Belgium) against the geometric mean of the three stably expressed housekeeping genes *G6PD*, *TBP*, *TUBA1A* scaled to d8 controls or to average values.

### 2.3. Immunofluorescence

For immunocytochemistry, hESCs were seeded on Matrigel-coated glass slides (SPL Life Sciences) and subjected to differentiation. The cells at day 8 (d8) were fixed in 4% (*w*/*v*) paraformaldehyde for 10–20 min at 4 °C and subsequently blocked for 20 min in PBS plus 0.2% Triton X-100 with 5% donkey serum (Dianova, Hamburg, Germany) and 1 mg/mL NaBH_4_. Primary and secondary antibodies were diluted in PBS with 0.1% Triton X-100 and 0.1% donkey serum. Primary antibodies were incubated overnight at 4 °C. Secondary antibodies were diluted 1:500 and incubated for 1 h at room temperature. The primary antibodies anti-HNF1B (SantaCruz, sc-22840, Santa Cruz, CA, USA) and anti-PDX1 (R&D systems, AF2419, Minneapolis, MN, USA) were used. Secondary antibodies were obtained from Dianova (Hamburg, Germany) conjugated with AlexaFluor or Cy fluorophores. Finally, the slides were mounted with Immunoselect antifading mounting medium containing DAPI (Dianova). Stained cells were examined using an Olympus IX81 microscope (Olympus, Tokyo, Japan). For PDX1-positive cell counting, between 6–10 pictures for each condition from 3 experiments were taken and the cells were quantified using the plugin Image-based Tool for Counting Nuclei (ITCN) on Image J.

### 2.4. SHH-ELISA

Media supernatants from d0, d4, and d8 of differentiation were collected, centrifuged at 2000× *g* for 5 min and in the resulting medium supernatants the human SHH protein concentration was assessed using the Quantikine ELISA hSHH kit (R&D systems) against a standard curve from 0 to 2000 pg/mL human SHH.

### 2.5. SHH Activity Assay

HEK293FT cells were electroporated with different combinations of pGL4.75 (*renilla firefly*, transfection control), the SHH reporter plasmids 8x3’Gli-BS-delta51-LucII, 8xm3’Gli-BS-delta51-LucII, 8xm3’Gli-BS-delta51-LucII and the human Gli1 encoding plasmid pcDNA3.1-His-hGLI1 [16]. After 24 h the cells were incubated with media supernatants from hESC differentiation cultures collected from controls and FGF2-treated cells. After further 24 h a dual luciferase assay was performed on a GloMax microplate reader using the dual luciferase assay system (Promega).

### 2.6. Transcriptomics

For transcriptomics, total RNA from three d0 and three d4 samples were pooled in equal proportions, reverse transcribed, and hybridized on a whole human genome oligo microarray 4x44K v2 (Design ID 026652, Agilent Technologies, (Santa Clara, CA, USA). Readout was performed on an Agilent Microarray Scanner G2565CA. Further details are summarized in the supplemental section (Supplementary methods).

### 2.7. Proteomics

Secretome analysis was performed as previously described [17]. Briefly, media supernatants conditioned for 24 h from HES3 grown in E8 medium (d0 samples) and from HES3-derived endodermal cells (d4 samples) grown in xenogeneic-free endoderm differentiation medium [13] were collected and centrifuged at 2000× *g* for 5 min to remove suspended cells and cell debris. A total of 8–9 mL of cell culture supernatants were then applied for precipitation according to the TCA-NLS method [18,19]. The samples were applied to SDS-PAGE using 5% stacking and 15% separating gels. After staining with Coomassie (Roti-Blue, Carl Roth, Karlsruhe, Germany), sample lanes were cut into pieces and digested with trypsin according to Shevchenko et al. [20]. Peptides were analyzed by liquid chromatography–mass spectrometry (LC–MS/MS) using a nanoflow ultrahigh pressure liquid chromatography system (RSLC, Thermo Fisher Scientific) for reversed-phase chromatography [17]. The RSLC system was coupled to an LTQ Orbitrap-Velos mass spectrometer by a Nano Spray Flex Ion Source II (Thermo Fisher Scientific). Raw data processing was performed with MaxQuant proteomics software suite version 1.4.1.2. Andromeda [21] was used for analysis of the peak lists against the entries of human proteins in the UniProt database (database downloaded on 9 July 2015) and relative amounts of proteins were determined by label-free quantification. Perseus (Version 1.1.6.1.3) was used for imputation (operation “replace missing values from normal distribution”), statistical analysis including *t*-tests, Pearson’s correlation, and Principal Component Analysis (PCA), for the implementation of the transcriptome dataset, and the annotation of belonging to selected GO terms [22]. Protein levels were compared quantitatively based on label-free quantification (LFQ) intensities and candidate proteins were selected according to our search parameters GO-terms 005125, 007173, 0008543, 0008083, 0048012, 0005179, 0038092, 0007219, 0048384, 0007224, 0007179, 0038084, and 0016055 (Supplementary Table S3).

### 2.8. Western Blot

Cells at d4 were incubated with stage 2 medium supplemented with 10 or 50 ng/µL FGF2. After 10, 20 and 30 min the cells were detached, collected, centrifuged, and resuspended in RIPA buffer (Thermo Fisher Scientific). Whole cell extracts were sonified and a protease inhibitor mixture (Roche Diagnostic) was added. The protein content was determined by BCA assay (Thermo Fisher Scientific). 20 µg of total protein was separated on two gels by SDS-PAGE and transferred by electro-blotting to a PVDF membrane. Blocking was performed with 5% nonfat dry milk in PBS plus 0.1% Tween 20. The primary antibodies anti-pJNK (#4671, Cell Signaling Technology, Danvers, MA, USA), anti-pERK (Santa Cruz, sc16982, anti-p-p38 (Cell Signaling Technology, 4631/9211) and anti-actin (Santa Cruz, sc1615) were incubated overnight at 4 °C, washed and incubated with the peroxidase-labeled secondary antibodies for 1 h. Protein bands were visualized by chemiluminescence using ECL select or ECL detection kit (GE Healthcare Europe, Chicago, IL, USA) on a chemiluminescence imager (INTAS Science imaging, Göttingen, Germany). Densitometric measurements of two independent Western blots was performed with Image Studio Lite (LI-COR Biosciences, Lincoln, NE, USA).

### 2.9. Statistics

Unless stated otherwise, all data values represent means ± SEM. Statistical analyses were performed using the GraphPad Prism (version 5.0.3) or Perseus software (Version 1.1.6.1.3)

## 3. Results

### 3.1. Effect of Growth Factors on Early Pancreatic Development

**Figure 1:**
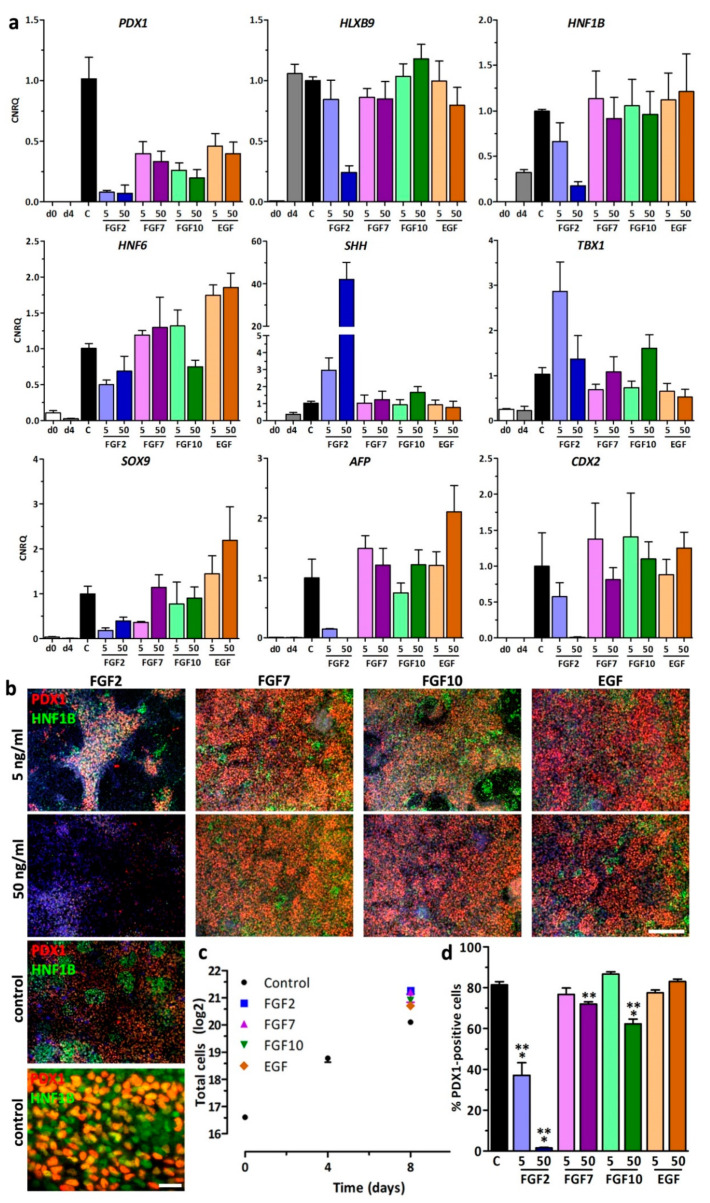
Pancreatic differentiation in presence of growth factors. (**a**) Gene expression of *PDX1*, *HLXB9*, *HNF1B*, *HNF6*, *SHH, TBX1, SOX9, AFP,* and *CDX2* measured by RT-qPCR in human embryonic stem cells (hESC)s at d0, d4, and d8 of differentiation treated ± 5 or 50 ng/mL fibroblast growth factor (FGF)2, FGF7, FGF10, or epidermal growth factor (EGF) in stage 2 medium. Values are means ± SEM, *n* = 4–6. 5/50 = 5 or 50 ng/mL growth factor, C = control medium without growth factors. (**b**) Fluorescence micrographs after pancreatic differentiation illustrating the protein expression of PDX1/HNF1B after a 4-day treatment ± 5 or 50 ng/mL FGF2, FGF7, FGF10, and EGF. Nuclei were counterstained with DAPI. Scale bar = 200 µm and 20 µm for higher magnification of the control. (**c**) Quantification of cell expansion using ± 50 ng/mL growth factor. Data are presented as mean (log2) ± SEM, *n* = 3. (**d**) Quantification of PDX1-positive cells upon treatment ± 5 or 50 ng/mL FGF2, FGF7, FGF10, and EGF at d8. Percentages are expressed as means ± SEM. *** = *p* ≤ 0.001, ** = *p* ≤ 0.01 compared to control, ANOVA plus Dunnett’s post-test.

After definitive endoderm induction for 4 days in stage 1 media, hESCs were incubated under control conditions in stage 2 medium, which induced the early pancreatic state hallmarked by PDX1 expression (Figure 1a,b). Cells at this stage represent posterior foregut cells and do not yet express NKX6-1. Importantly this stage 2 medium lacks growth factors but contains only human recombinant insulin as a mitogen. To test the ability of growth factors to effect the expansion and differentiation during stage 2, the medium was supplemented with low (5 ng/mL) and high concentrations (50 ng/mL) of FGF2, FGF7, FGF10, and EGF (Figure 1a,b). After 4 days the cells were analyzed for expression of the early pancreatic genes *PDX1*, *SOX9*, *HLXB9*, *HNF1B*, *AFP*, *CDX2*, and *HNF6* and two genes, *SHH* and *TBX1*, which we previously found to be substantially induced by FGF2 during anterior–posterior patterning of the definitive endoderm [12]. *AFP* and *CDX2* were tested to assess the ability of growth factors to direct DE cells into other lineages than that of the pancreas.

RT-qPCR analysis revealed the induction of pancreatic genes during stage 2 in controls and after supplementation with FGF7, FGF10, and EGF with small differences with respect to the growth factor and its concentration. Surprisingly, *PDX1*, *HLXB9*, *HNF1B*, *HNF6, AFP* and *CDX2* gene expression was suppressed by FGF2 whereas expression of *SHH* and *TBX1* was markedly increased. *SOX9* gene expression was also mildly lowered in presence of FGF2 (Figure 1a).

This result was confirmed by immuno-fluorescence analysis, which showed a nuclear localization of PDX1 and HNF1B in all conditions except in FGF2-treated cells. Already low concentrations of FGF2 yielded in a substantial decrease in PDX1/HNF1B double-positive cells and complete loss of PDX1/HNF1B was detected for high FGF2 concentrations (Figure 1b). Measurement of cell expansion after 8 days showed >10-fold cell expansion under control conditions and 25-fold, 24-fold, 20-fold, and 17-fold cell expansion for FGF2, FGF7, FGF10, and EGF, respectively (Figure 1c). The quantification of PDX1-positive cells at d8 affirmed the gene expression result. Around 81% PDX1-positive cells were counted for the control condition. Although control cells showed the highest PDX1 gene expression, the amount of PDX1-positive cells was not significantly higher than in cells treated with low FGF7, low FGF10 and low/high EGF. FGF2 decreased PDX1-positive cells by 50% at low concentrations and almost 100% at high concentrations. Other treatments with growth factors yielded results comparable to controls. Surprisingly high concentrations of FGF7 and FGF10 were also slightly detrimental to pancreatic lineage progression (Figure 1d).

**Figure 2:**
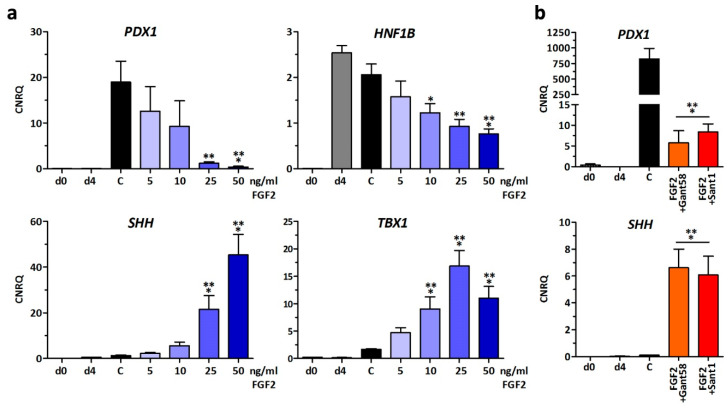
Gene expression changes in response to different FGF2 concentrations. (**a**) hESCs were cultured in the presence of 5–50 ng/mL FGF2 during differentiation into PDX1-positive cells and compared to a control condition (=C). Gene expression changes of *PDX1*, *HNF1B*, *SHH,* and *TBX1* are presented as means ± SEM, *n* = 5–12. *** = *p* ≤ 0.001, ** = *p* ≤ 0.01, * = *p* ≤ 0.05 compared to control, ANOVA plus Dunnett’s post-test. (**b**) Effect of SHH inhibition by 10 µM Gant 58 or 2.5 µM Sant1 in stage 2 medium ± 50 ng/mL FGF2. Gene expression changes of *PDX1* and *SHH* are presented as means ± SEM, *n* = 4–5. *** = *p* ≤ 0.001, compared to control, ANOVA plus Dunnett’s post-test.

Next, dose-dependency of FGF2 during stage 2 was tested. A concentration dependent loss of *PDX1* and *HNF1B* gene expression was observed. Alternatively, *SHH* and *TBX1* gene expression was significantly increased (Figure 2a). Based on these observations, we hypothesized that FGF2 triggers the expression of the anti-pancreatic signaling molecule *SHH*, resulting in an augmented secretion of SHH and autocrine inhibition of pancreatic specification. Thus, we initially attempted to chemically inhibit the SHH pathway. SHH is a known anti-pancreatic signaling molecule [3] and thus we hypothesized that the highly induced SHH gene expression would result in an increased secretion of SHH protein into the stage 2 medium that, in an autocrine fashion, might inhibit pancreatic lineage progression. However, SHH inhibition by Sant1 (inhibits SMO) and Gant58 (Gli2 inhibitor) in presence of FGF2 did not rescue PDX1 nor reduce SHH gene expression (Figure 2b).

### 3.2. Small Molecule Inhibition of FGF-Signaling

**Figure 3:**
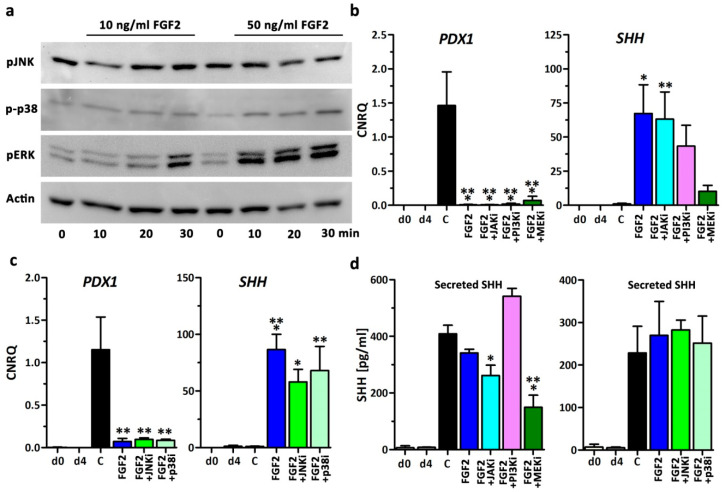
Small molecule inhibition of FGF2-signalling. (**a**) Analysis of JNK, ERK and p38 protein phosphorylation by Western blot after incubation of definitive endoderm cells at d4 cultured in stage 2 medium supplemented with 10 or 50 ng/µl FGF2 for 0, 10, 20, and 30 min, respectively. (**b**,**c**) Gene expression changes of *PDX1* and sonic hedgehog (*SHH)* at d8 after treatment with 50 ng/mL FGF2 and small molecule inhibitors of Janus kinase/signal transducer and activator of transcription (JAK/Stat), PI3K, MEK, JNK, and p38. Values are means ± SEM, *n* = 3–5. C = control without growth factors. ** = *p* ≤ 0.001, ** = *p* ≤ 0.01, * = *p* ≤ 0.05 compared to control, ANOVA plus Dunnett’s post-test. (**d**) Measurement of secreted SHH in the media supernatants by ELISA. The cells were differentiated with 50 ng/mL FGF2 plus small molecule inhibitors of PI3K-, JAK/Stat1-, MEK/ERK-, JNK-, and p38-signaling to block the FGF signaling downstream of the receptor. Data are presented as means ± SEM, *n* = 3–6 and represent the secretory capacity of 24 h. *** = *p* ≤ 0.001, * = *p* ≤ 0.05 compared to control, ANOVA plus Dunnett’s post-test.

Next the possibilities of suppressing the downstream transduction cascades of the FGF receptor were assessed. FGF-signaling downstream of the tyrosine kinase receptor can be transduced by four major pathways to the nucleus and comprises: JAK/STAT (Janus kinase/signal transducer and activator of transcription), PLCγ and PKC (phosphoinositide phospholipase C and phosphatidylinositol 3-kinase isoform C) and two MAPKinase pathways (mitogen-activated protein kinases) yielding in phosphorylation of p38, JNK, and ERK1/2, respectively [23]. Western blot analysis of these kinases revealed that definitive endoderm cells at d4 exposed to stage 2 medium supplemented with either 10 or 50 ng/mL FGF2 increased phosphorylated ERK1/2 and p38 but not JNK (Figure 3a). Densitometric Western blot quantifications are presented in Supplementary Figure S1.

Thus, in two sets of independent experiments, small molecule inhibitors of JAK/STAT (JAKi), PI3Kinase (PI3Ki), MEK/ERK (MEKi), JNK (JNKi), and p38 (p38i) were additionally supplemented to FGF2-containing stage 2 medium and compared to controls (Figure 3b,c). Inhibition by small molecule inhibitors did not rescue *PDX1* gene expression to a level comparable to controls. However, *SHH* gene expression was markedly downregulated by MEKi and slightly by PI3Ki. Other inhibitors showed no effects (Figure 3b,c). Then we measured the amount of secreted SHH in the supernatant (stage 2 medium) of differentiated cells by ELISA (Figure 3d). HESCs at d0 and definitive endoderm at d4 did not secret substantial amounts of SHH to the medium. Surprisingly at d8, control cells and cells exposed to FGF2-supplemented medium showed a distinct and similar concentration of 410 pg/mL (control) and 341 pg/mL (FGF2) SHH protein in the culture medium (Figure 3d). Chemical inhibition of the signaling cascades showed a significant decrease in SHH secretion by MEKi and to a lower degree by JAKi. Thus, we concluded that SHH gene expression and secretion in endoderm cells depends on the MEK/ERK-signaling cascade. In order to assess whether secreted SHH is biologically active and may inhibit further pancreatic differentiation, we used a specific SHH reporter assay [16]. HEK293FT cells were transiently transfected with a sensitive SHH plasmid-based reporter gene system, a transfection control, and a plasmid encoding human Gli1 as an assay control (Supplementary Figure S2). After 24 h the reporter cells were subjected to conditioned medium from control cells or cells exposed to FGF2-supplemented medium. Measurement of the firefly luciferase activity revealed no substantial increase when comparing FGF2 against controls and when comparing to cells transfected with plasmids containing mutated Gli1 binding sites (Supplementary Figure S2). Hence the concentration of secreted SHH in the medium is either too low or not biologically active to antagonize pancreatic development in this in vitro system.

### 3.3. Secretome Analysis

Intrigued by the finding that developing hESCs actively contribute to the spatio–temporal milieu of growth factors in the differentiation medium, we continued to assess the secretory profile of hESCs prior to pancreatic induction. For that purpose hESCs were cultured in BSA- and serum-free TeSR-E8-medium and subsequently differentiated in chemically defined stage 1 medium [13,17]. Media supernatants from d0 and d4 were collected and quantitatively analyzed by LC-MS/MS. The resulting dataset was additionally integrated with whole genome transcriptomics. Supplementary Figure S3 illustrates the workflow of data processing with the Perseus software. In summary, secretome analysis led to the identification of 3819 proteins. Common contaminants were removed and we applied stringent selection parameters: proteins must be identified at least by two peptides in at least three of the four replicates for at least one time point. This yielded in 2708 detected proteins. From this dataset, 45 and 9 proteins were exclusively present at d0 or at d4 (Supplementary Table S2).

**Figure 4:**
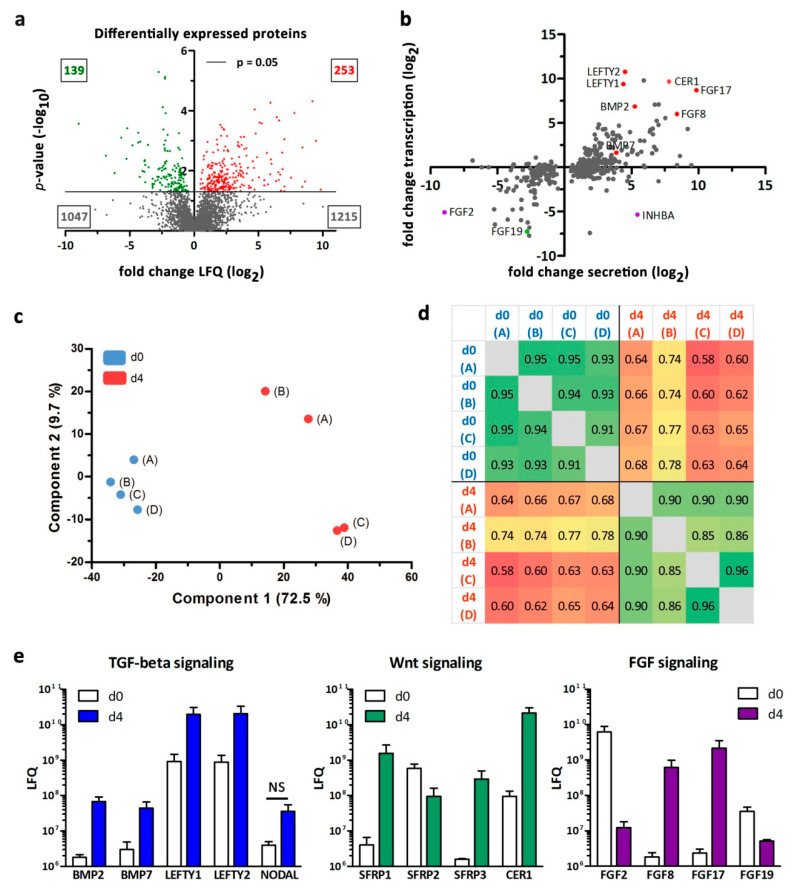
Assessment of secreted proteins by mass spectrometry. (**a**) Volcano plot of 2654 proteins (log2 of label-free quantification (LFQ) fold changes) identified in d0 and d4 cells. A total of 392 proteins were significantly altered (*p* ≤ 0.05, 2 sample Student’s *t*-test). A total of 139 proteins were downregulated (green) and 253 proteins were significantly upregulated (red) in d4 compared to d0 samples. (**b**) Correlation of fold change secretion changes (log2) with fold change transcription changes (log2) in proteins/genes significantly altered at d4 compared to d0. Candidate genes from selected signaling pathways are highlighted. (**c**) Principal component analysis (PCA) of the 392 significantly altered proteins for four individual samples (A–D) at d0 (blue) and d4 (red), respectively. (**d**) Pearson correlation for four biological replicates (A–D) at d0 and d4, respectively. (**e**) Mean LFQ values of relevant secreted proteins in supernatants of d0 and d4 cells samples belonging to TGF-beta-, Wnt-, and FGF-signaling pathways. All proteins, except NODAL, were significantly changed (*p* ≤ 0.05, two sample Student’s *t*-test) (SFRP3 = FRZB).

After log2-transformation and imputation of missing values for the 2654 proteins that were found in both datasets (at d0 and d4), statistical analysis revealed 392 significantly altered proteins with *p* ≤ 0.05 of which 253 and 139 were significantly up- and down-regulated at d4, respectively (Figure 4a). Fold changes of protein levels that were statistically significantly different between d0 and d4 were plotted against the respective fold changes observed on transcriptional level (Figure 4b). A set of candidate factors with pro- or anti-pancreatic activity are highlighted in Figure 4b including several members of the FGF-, TGF-beta-, and Wnt-family. Notably, FGF2 was supplemented into the TeSR-E8 medium (d0) and activin A (INHBA) was supplemented into the stage 1 medium (d4). Accordingly, high levels of FGF2 and INHBA were detected at day 0 and d4, respectively, which confirmed our analytical approach but prevented the identification of endogenous FGF2 and INHBA levels (Figure 4b). Principal component analysis (PCA) and Pearson’s correlation analysis of the d0 and d4 samples showed clustering of the four replicates A-D and less correlation for the different time points (Figure 4c,d). Absolute LFQ-intensities were displayed for selected secreted factors belonging to the TGF-beta-, Wnt- and FGF-signaling pathways (Figure 4e). The relevant receptors, with the exception of *BMPR1B* were also identified in the transcriptome dataset in d0 and d4 samples (Supplementary Figure S4). In comparison to d0 cell culture supernatants, d4 media are conditioned with highly increased levels of the BMP signaling agonists BMP2 and BMP7, the nodal signaling antagonists LEFTY1 and 2, the Wnt signaling antagonists SFRP1 and SFRP3 (FRZB), the multipurpose antagonist Cerberus, and the FGF-family members FGF8 and FGF17 (Figure 4e, Table 1). LFQ-intensity of the TGF-beta agonist Nodal, whose activity is necessary for definitive endoderm formation, was also augmented on d4 compared to d0 but these changes missed the significance threshold. The list of 392 proteins that were significantly altered between d4 and d0 was searched against selected terms of gene ontology (GO) with growth factor, hormone, or cytokine activity or affiliation to signaling pathways relevant for early pancreatic development. This led to the identification of 41 proteins of which 29 were up-regulated and 12 down-regulated on d4 (Supplementary Tables S1 and S2).

### 3.4. FGF-Signaling through FGFR1c/3c

**Figure 5:**
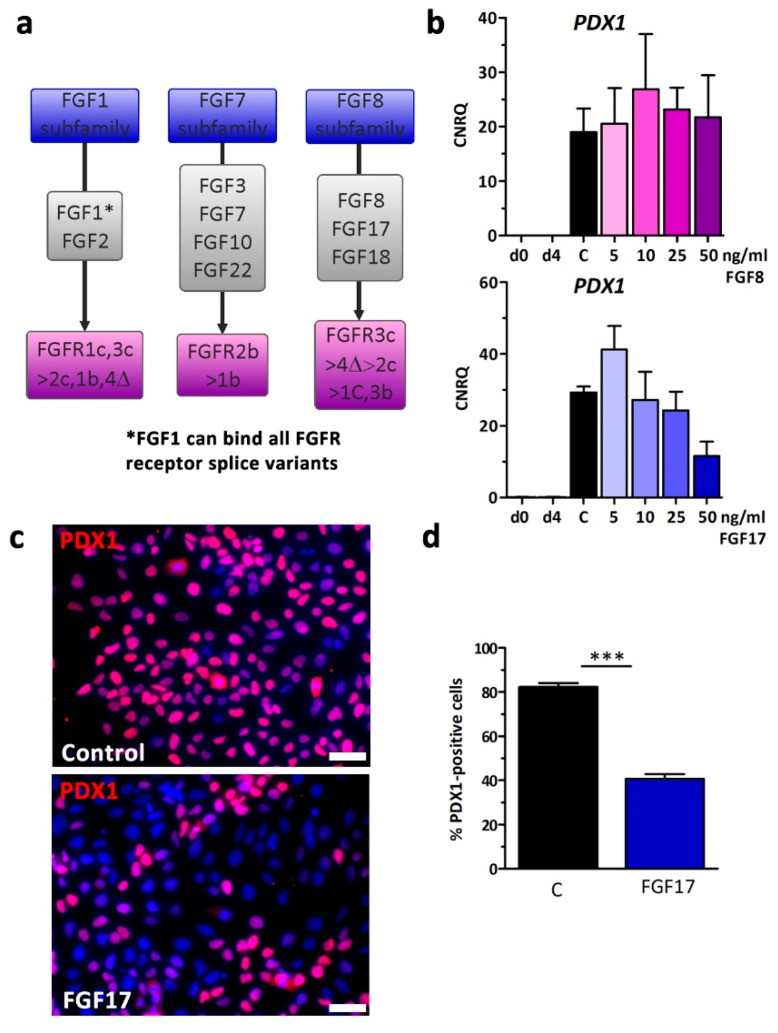
FGF-signaling through FGFR1c/3c inhibits pancreatic development. (**a**) Schematic presentation of ligand and receptor specificities of selected FGF family members [24,25]. (**b**) hESCs were subjected to 5–50 ng/mL FGF8 or FGF17 during differentiation into PDX1-positive cells compared to control without growth factors. The gene expression change of PDX1 is presented as means ± SEM, *n* = 3–4. (**c**) Representative fluorescence micrographs after pancreatic differentiation illustrating the protein expression of PDX1 after a 4-day treatment ± 50 ng/mL FGF17. Nuclei were counterstained with DAPI. Scale bar = 50 µm. (**d**) Quantification of PDX1-positive cells at d8. Data are means ± SEM, *n* = 3. *** = *p* ≤ 0.001, two-tailed Student’s *t*-test.

Two of the most highly up-regulated growth factors in the d4 medium supernatant, FGF8 and FGF17, belonged to the FGF8 subfamily (Table 1). Strikingly, FGF8 and FGF17 share receptor specificity with FGF2 and predominantly bind the receptor splice variants FGFR3c and potentially also FGFR1c (Figure 5a) [24].

**Figure 6:**
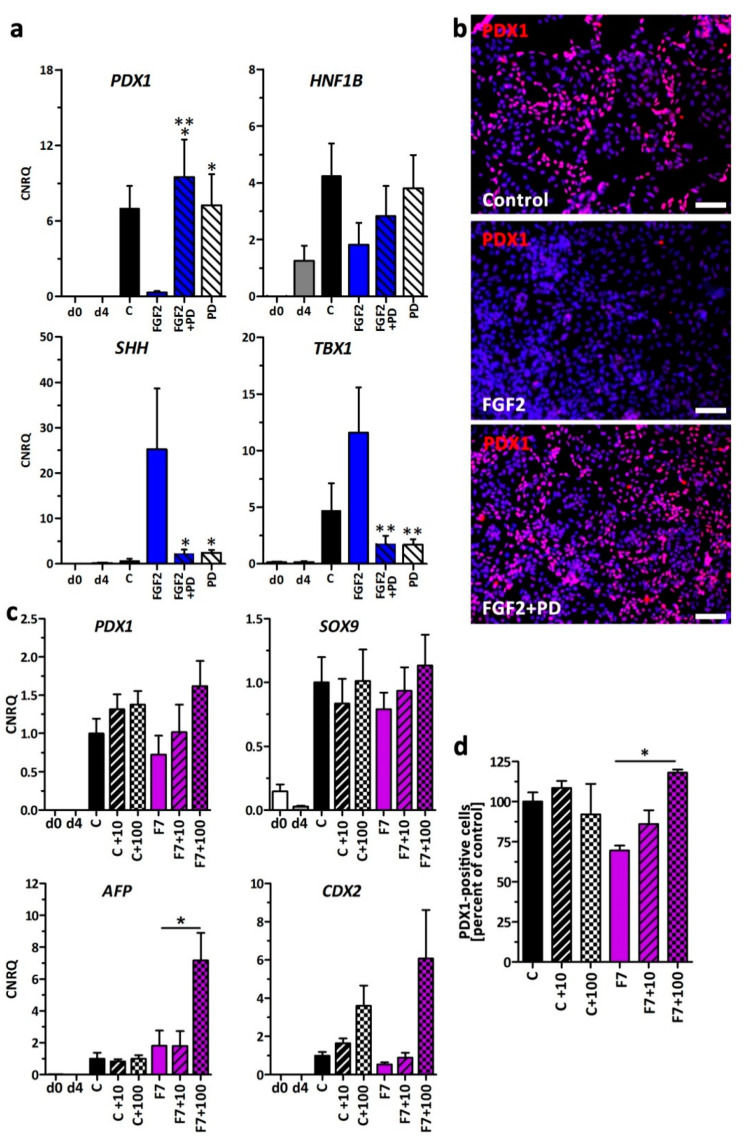
Inhibition of FGFR1c/3c rescues pancreatic development in presence of FGF2 and FGF7. (**a**) Gene expression changes of *PDX1*, *HNF1B*, *SHH*, and *TBX1* in d8 cells treated ± 10 ng/mL FGF2 (F2) and the FGFR1c/3c small molecule inhibitor PD-173074 (PD) with a concentration of 100 nM (PD). Data are means ± SEM, *n* = 4–5. *** = *p* ≤ 0.001, ** = *p* ≤ 0.01, * *p* = ≤0.05 ANOVA plus Tukey’s post-test, F2 treatment compared to F2 + PD and PD only. (**b**) Representative immunofluorescence staining of PDX1 in d8 control cells, cells treated with F2 and F2 + PD. Nuclei were counterstained with DAPI. Scale bar = 100 µm. (**c**) Gene expression changes of *PDX1*, *SOX9*, *AFP*, and *CDX2* in d8 cells treated ± 50 ng/mL FGF7 (F7) and ± PD-173074 with a concentration of 10 or 100 nM (10, 100). Values are means ± SEM, *n* = 3–6, * = *p* ≤ 0.05, ANOVA plus Tukey’s post-test. (**d**) Quantification of PDX1-positive cells at d8 upon treatment ± 50 ng/mL FGF7 and ± PD-173074 with a concentration of 10 or 100 nM. Percentages are expressed as means ± SEM. * = *p* ≤ 0.05, ANOVA plus Tukey’s post-test.

Thus, we supplemented stage 2 medium with 5–50 ng/mL FGF8 and FGF17 and measured the effects on *PDX1* expression (Figure 5b,c). Compared to control FGF8 was not able to reduce *PDX1* expression, however, FGF17 reduced *PDX1* in a dose-dependent manner, comparable to FGF2 but less pronounced (Figure 2a). PDX1 was also found to be reduced in immunofluorescence staining. Image quantification revealed a significant 50% loss from 81% in controls down to 41% PDX1-positive cells when FGF17 was supplemented to the differentiation medium at 50 ng/mL (Figure 5d). We speculated that FGFR3c might be responsible for the suppressive anti-pancreatic effect and used the selective FGFR1c/3c inhibitor PD-173074 (PD, IC_50_ = of 5 nM and 21.5 nM, for FGFR1c and FGFR3c, respectively) [26], attempting to rescue the effect of FGF2. Briefly, stage 2 medium was supplemented ± FGF2, FGF2 + PD, or PD alone and then FGF2-affected genes were measured by RT-qPCR (Figure 6a).

By treating the cells with PD the expression of *PDX1* and *HNF1B* could be rescued whereas *TBX1* and *SHH* were suppressed to levels near control (Figure 6a). PD alone showed a moderate cytotoxicity to the cells (data not shown). Immunofluorescence staining of PDX1 confirmed the gene expression result. PDX1 was readily detected in control cells and cells treated with FGF2+PD (Figure 6b). FGF7 along with FGF10 is the most commonly used growth factor in beta cell differentiations. We found a mild reduction of PDX1-positivity when this growth was used in its typical concentration of 50 ng/mL (Figure 6d). To address the question whether FGFR1c/3c inhibition is also able to abrogate this effect, we differentiated the cells ± 50 ng/mL FGF7 and ± 10 or 100 nM PD-173074. FGFR1c/3c inhibition yielded in a mild increase in *PDX1* and *SOX9* gene expression in FGF7-treated cells. Surprisingly gut (*CDX2*) and liver (*AFP*) marker genes were also upregulated (Figure 6c). Finally, we quantified the number of PDX1-positive cells and could show that FGFR1c/3c inhibition was able to significantly increase PDX1 protein expression in presence of high FGF7 concentrations (data expressed as percentage of controls, Figure 6d).

## 4. Discussion

Signaling molecules, such as retinoic acid (RA), activin-βB, EGF, FGF2, FGF7, and FGF10, released from the mesoderm to the endodermal epithelium of the primitive gut tube are required for the initiation of pancreatic progeny. To explore this mechanism in the human system, we analyzed the effects of the exogenously supplied growth factors EGF, FGF2, FGF7, and FGF10 during the in vitro differentiation of hESC-derived DE into early pancreatic–duodenal PDX1-positive cells.

PDX1 expression, which hallmarks early pancreatic–duodenal progeny, was chosen as our main marker along with HNF6 (Oncecut1), HNF1B and HLXB9. Differentiation of hESC-derived DE using a growth factor-free control protocol [12] resulted in a substantial number of PDX1-positive cells. Additional supplementation with growth factors enhanced the cell mass at this early differentiation stage. This is in line with previous findings in rodents [5,6] where EGF, FGF7, and FGF10 enhance proliferation of explanted embryonic pancreases. Regarding the highest PDX1 gene expression in control cells we assume that the expression of PDX1 is on average per cell slightly higher compared to in low FGF7-, low FGF10-, or EGF-treated cells.

FGF2 secreted by the notochord and dorsal aortae is thought to be a significant contributory factor suppressing SHH activity in the posterior foregut endoderm of the primitive gut tube, thereby permitting the development of the pancreas anlagen [4]. Thus, FGF2, FGF7, FGF10, and EGF are routinely used in protocols for the expansion of hESC-derived DE and the PDX1-positive pancreatic endoderm [8,9,10,11,27]. Under our conditions FGF7, FGF10, and EGF substantially increased the cell mass in vitro while retaining pancreatic identity. Surprisingly FGF2, as opposed to the mechanism described for chick [3], suppressed pancreatic lineage progression and effectively induced *SHH* and *TBX1* gene expression.

SHH produced and secreted by the endodermal epithelium of the primitive gut tube is known to control smooth muscle development in the surrounding mesoderm [2]. Hence, it is not surprising that we found SHH expressed and secreted to the medium of the control. FGF2 supplementation strongly induced SHH gene expression. However, we did not observe any further increase in the medium concentration of SHH upon treatment with FGF2. This mechanism remains unclear in this study and will certainly require further experimentation in the future. However, supportive to our findings, the FGF2–SHH axis is described for limb development in mice where FGF2 maintains SHH expression and SHH, in turn, maintains FGF2 [28]. Combined treatment of hESC-derived endodermal cells (d4) with FGF2 and small molecule inhibitors of the FGF receptor pathways brought evidence that SHH expression is induced via MEK/ERK signaling. However, pharmacologic inhibition of the MEK/ERK- and SHH-pathway did not restore PDX1 gene expression to the level of the controls.

FGF2 supplementation caused increased expression of *TBX1*, which is indicative of a definitive endoderm primed towards pharyngeal endodermal progeny. *TBX1* is expressed along with *FGF8* in the endoderm anterior of the stomach [29]. Thus, FGF2 may shift cellular identify from the posterior foregut endoderm towards the anterior foregut endoderm. Alternatively, there is evidence that FGF2 induces *TBX1* expression during odontogenesis [30]. The detected increase in *TBX1* in FGF2-treated DE may reflect this regulatory relationship although in an artificial manner.

A number of studies have already reported that developing hESC/iPSC contribute to a paracrine microenvironment in the culture medium by secretion of growth factors and cytokines [17,31]. The differentiation of hPSCs into stem cell-derived insulin-producing beta cells is often hindered by low consistency [32]. As such we wondered whether the secretome of the cells prior to pancreatic induction, namely at the stage of DE, comprises factors either detrimental or advantageous to pancreatic lineage progression. Proteomic data during hPSC pancreatic differentiations were recently published [33], however we opted for a secretomic analysis to identify only actively secreted factors.

Our first-time time data on the secretome of hESC and DE cells revealed a substantial number of proteins present in media supernatants. Almost 300 proteins were significantly higher or exclusively secreted at d4, whereas only ~150 proteins were more highly abundant at d0. Thus, gain of cellular function and differentiation status resulted in an increased secretory activity of the cells. Naturally not all detected proteins might have been actively secreted. In vitro cultured cells typically suffer from apoptosis, necrosis, and hydrodynamic shear force, causing release of cytoplasmic proteins to the culture medium. Thus, we compared the d4 to d0 secretome and applied stringent GO-term filtering to narrow down the protein lists to actively secreted growth factors and cytokines with putative functions during pancreas development. Whole transcriptome data served as controls to retrieve only differentially expressed genes with clear-cut correlation to the secretome. Proteins of the TGF-beta-, Wnt- and FGF-signaling pathways were up-regulated in the d4 secretome.

LEFTY1, LEFTY2, and NODAL are members of the TGF-beta pathway. Previously it could be shown that all three are expressed in undifferentiated hESCs [17,34]. LEFTY proteins function as antagonists to activin A/Nodal-signaling to organize left–right symmetry in the embryo [35]. LEFTY proteins also add to spatial differences confining NODAL expression to the posterior pre-gastrulation embryo defining the space for the mesendodermal precursor population [35]. Nodal-signaling can trigger LEFTY gene expression [36]. Thus, the high concentrations of LEFTY proteins may reflect the result of the hyperphysiological stimulation of hESC with activin A during the first 4 days of in vitro differentiation. Vice versa, high LEFTY concentrations in the medium may increase the need for further activin A supplementation in order to reach satisfactory differentiation efficiencies. Regarding further pancreatic specification, the literature does not record any specific role for LEFTY1 or LEFTY2 as TGF-beta signaling becomes important only at later stages of pancreas development [1].

BMPs also belong to the TGF-beta superfamily. BMP7 and BMP2 were detected in the secretome. BMP7 comprises manifold functions during kidney development and kidney disease but no described pro- or anti-pancreatic effect [37]. On the contrary, BMP2 is a known morphogen that can direct developmental cues away from the pancreas towards liver development [38]. BMP2 was already detectable at d0 and then LFQ-values increased almost 40-fold in d4 cells. Remarkably, BMP2 and Wnt-signaling inhibitors are routinely used during hepatic differentiation of hESC-derived DE [39].

CER1 can antagonize BMP, NODAL and Wnt-signaling. Kempf and colleagues proposed a model in which Wnt- and BMP-signaling drive primitive streak progression along the anteroposterior axis from definitive endoderm to presomitic mesoderm, which can be antagonized by endogenous LEFTY1 and CER1 [31]. However, the multifaceted nature of CER1 renders its potential role during pancreatic in vitro differentiation difficult to predict.

Four out of 22 members of the human FGF family have been identified in our secretome dataset. FGF2 was used as supplement of the stem cell maintenance medium and was accordingly detected at high levels at d0 and decreased at d4. The same pattern was observed for FGF2 gene expression. Although we cannot discriminate between exogenously added and endogenously secreted FGF2 by proteomics, our expression data indicate that d0 and d4 cells actively produce and thus secrete FGF2 to the supernatant. FGF19 was also present in the d0 supernatant and declined upon differentiation. FGF19 expression and protein secretion has already been described for human pluripotent cells, although the function of FGF19 in this context remains unclear [17,40]. FGF8 and FGF17 levels were highly increased at d4 in comparison to d0 with LFQ-values >10^9^ indicating biologically relevant concentrations. FGF8 expression has been associated with endoderm identity anterior of the stomach [29] suggesting that DE on d4 contains anterior foregut endoderm cells. Surprisingly, secreted FGF8 was also detected in our previous study in early cardiac mesoderm suggesting multiple roles of this growth factor during in vitro gastrulation of hESCs/hiPSCs [17]. Notably, FGF17 showed >900-fold increased levels in cell culture supernatants comparing d4 to d0. FGF17 has no known function during pancreas development but surprisingly FGF8, FGF17, and FGF2 share the same specificity for binding and activation of the FGF receptor splice variants FGFR3c and FGFR1c [24]. Supplementation of stage 2 medium with FGF8 and FGF17 brought evidence that only FGF17 exerted anti-pancreatic effects comparable to FGF2. Taken together, we conclude that the hESC-derived endoderm secretes paracrine factors into the cell culture medium that act in an anti-pancreatic manner.

Rescue attempts targeting the individual branches of the FGF pathway to restore PDX1 gene expression have been so far unsuccessful. A likely explanation is that small molecule inhibition within the signaling pathway is less efficient and is outperformed by the amplifying nature of the signaling cascade. Thus, a reasonable approach to tackle this issue could be to target the receptor itself. We used the specific FGFR1c/3c inhibitor PD-173074 [26], to restore PDX1 gene expression. Thereby we could show that the anti-pancreatic–duodenal impact of FGF2, which was deduced from suppression of PDX1 and induction of *SHH* and *TBX1*, is mediated by the receptor splice variants FGFR1c/3c. We conclude that for humans FGF2/FGF17 inhibit pancreatic differentiation through binding to FGFR1c/3c, whereas proliferation and suppression of further differentiation, as reported for rodents, is mediated by FGFR2b with its ligands FGF7 and FGF10 [5,6].

It is possible that our findings mirror the mechanism described for the ventral pancreatic bud formation in mice where FGF2 secreted from the cardiac mesoderm induced hepatogenesis at the expense of pancreatic development [41]. Thus, we measured *AFP* gene expression and found *AFP* was also suppressed by FGF2. This indicates a broader inhibitory role of FGF2 at this specific stage of posterior foregut cells.

Regarding previous findings for the human system we can refer to several studies using hESCs/hiPSCs. EGF has been shown to efficiently promote PDX1/NKX6.1-positive MPCs when combined with BMP inhibition and nicotinamide [11]. FGF7 and FGF10 are routinely used early in the differentiation protocol for the generation of stem cell-derived beta cells to increase PDX1-positivity [8,9,42,43]. According to our data this appears deliberate to increase the numbers of PDX1-positive cells during in vitro differentiation.

Two further studies reported the use of FGF2 during anterior-posterior patterning of DE into the PDX1-positive posterior foregut endoderm and pancreatic endoderm [10,27]. However, our findings hint towards a different role of FGF2 during pancreatic development by showing that FGF2 directs the development away from pancreatic–duodenal progeny.

In summary we have analyzed the effects of growth factors during early pancreatic development and have identified FGF2 as a potent anti-pancreatic–duodenal factor. FGF2 decreased gene and protein expression of PDX1 and further early pancreatic genes and increased SHH and TBX1. However, SHH expression and presence in the medium supernatant is no obstruction for pancreatic lineage. Chemical inhibition of the FGFR1c/3c receptor completely abolished FGF2-induced effects and may be helpful to foster PDX1 expression also in presence of other growth factors. Interrogation of the secretome during differentiation revealed a complex micro-milieu of signaling molecules. Amongst them we detected FGF17 exerting similar effects as FGF2 probably mediated by the receptor FGFR1c/3c.

Taken together, our secretome dataset will contribute to the understanding of tissue interactions during human development. The detection of anti-pancreatic signaling factors in the differentiation medium leads to important implications for in vitro differentiation protocols such as careful considerations of medium volume per cavity, medium change routines, and cell densities in order to minimize paracrine effects of signaling molecules actively secreted by developing hPSCs.

## Figures and Tables

**Table 1:** Secreted signaling factors in hESCs and definitive endoderm (DE) cells.

Gene Names	Protein Names	Mean LFQ (d0)	Mean LFQ (d4)	Fold Change LFQ (d4/d0)	Fold Change Transcription (d4/d0)
FGF17	Fibroblast growth factor 17	2.3 × 10^6^	2.2 × 10^9^	918.56	418.27
SFRP1	Secreted frizzled-related protein 1	4.0 × 10^6^	1.6 × 10^9^	392.12	2.43
FGF8	Fibroblast growth factor 8	1.8 × 10^6^	6.2 × 10^8^	338.51	64.36
CER1	Cerberus	9.6 × 10^7^	2.1 × 10^10^	223.01	816.30
SFRP3 (FRZB)	Secreted frizzled-related protein 3	1.6 × 10^6^	2.9 × 10^8^	185.39	46.72
DKK1	Dickkopf-related protein 1	1.3 × 10^6^	1.7 × 10^8^	129.79	27.65
FLRT3	Leucine-rich repeat transmembrane protein FLRT3	2.6 × 10^6^	3.0 × 10^8^	117.66	38.37
STC1	Stanniocalcin-1	2.9 ×10^7^	2.5 × 10^9^	85.60	18.76
NPPB	Natriuretic peptides	2.5 × 10^6^	1.5 × 10^8^	60.52	890.54
MDK	Midkine	1.7 × 10^7^	7.6 × 10^8^	43.79	2.77
INHBA	Inhibin beta A chain	6.0 × 10^6^	2.6 × 10^8^	43.67	0.02
BMP2	Bone morphogenetic protein 2	1.8 × 10^6^	6.9 × 10^7^	38.04	116.29
CPE	Carboxypeptidase E	9.5 × 10^7^	2.8 × 10^9^	29.74	15.76
LEFTY2	Left-right determination factor 2	8.9 × 10^8^	2.0× 10^10^	23.03	1757.26
LEFTY1	Left-right determination factor 1	9.2 × 10^8^	2.0 × 10^10^	21.16	679.87
GRN	Granulins	1.0 × 10^7^	1.7 × 10^8^	17.12	1.54
BMP7	Bone morphogenetic protein 7	3.0 × 10^6^	4.4 × 10^7^	14.70	3.16
NRP2	Neuropilin-2	1.1 × 10^7^	1.1 × 10^8^	9.73	0.40
ROR2	Tyrosine-protein kinase transmembrane receptor ROR2	1.3 ×10^7^	9.8 × 10^7^	7.31	19.48
ITGB5	Integrin beta-5	1.1 × 10^7^	7.4 ×10^7^	6.71	2.81
HSPB1	Heat shock protein beta-1	5.9 × 10^7^	2.7 × 10^8^	4.54	2.00
PTK7	Inactive tyrosine-protein kinase 7	2.9 × 10^8^	1.3 × 10^9^	4.54	1.10
NXN	Nucleoredoxin	5.7 ×10^7^	2.1 × 10^8^	3.64	1.70
GPC4	Glypican-4	6.7 × 10^8^	2.4 × 10^9^	3.63	1.61
NCBP1	Nuclear cap-binding protein subunit 1	2.5 × 10^7^	9.1× 10^7^	3.59	0.81
SNW1	SNW domain-containing protein 1	1.5 × 10^6^	5.0 × 10^6^	3.25	0.83
FKBP8	Peptidyl-prolyl cis-trans isomerase FKBP8	4.9 × 10^6^	1.4 × 10^7^	2.93	0.76
GTF2F2	General transcription factor IIF subunit 2	8.6 × 10^6^	1.9 × 10^7^	2.22	0.87
ADAM10	Disintegrin and metalloproteinase domain-containing protein 10	7.2 × 10^7^	1.5 × 10^8^	2.11	1.36
TIAL1	Nucleolysin TIAR	1.9 × 10^8^	1.1 × 10^8^	0.57	0.82
DDB1	DNA damage-binding protein 1	1.6 × 10^9^	7.4 × 10^8^	0.45	0.95
CTGF	Connective tissue growth factor	1.2 × 10^9^	4.7 × 10^8^	0.392	0.39
WDR12	Ribosome biogenesis protein WDR12	1.5 × 10^8^	4.3 × 10^7^	0.290	0.53
PARP1	Poly [ADP-ribose] polymerase 1	3.0 × 10^9^	5.8 × 10^8^	0.191	0.51

Red color = upregulated proteins, green color = downregulated proteins.

## Supplementary Materials

The following are available online at https://www.mdpi.com/2073-4409/9/9/1927/s1, Figure S1: Densiometric quantification, Figure S2: Biological activity of secreted SHH, Figure S3: Workflow of data processing using the Perseus software, Figure S4: Expression of receptors from the TGF-beta-, Wnt-, and FGF-pathway, Table S1: Primer pairs for gene expression analysis. Table S2: Proteins exclusively detected at d0 and d4. Table S3: GO-Term list.
