# Supplementary material for: FGF2 Inhibits Early Pancreatic Lineage Specification during Differentiation of Human Embryonic Stem Cells
Source: Cells. 2020 Aug 20;9(9):1927. doi: 10.3390/cells9091927 (PMC7565644; doi:10.3390/cells9091927)
Supplement: Supplementary file 1 [file cells-09-01927-s001.pdf]

Supplementary Methods

Microarray Experiments (Single Color Mode)

The Microarray utilized in this study represents a refined version of the Whole Human Genome Oligo Microarray 4 × 44K v2 (Design ID 026652, Agilent Technologies, (Santa Clara, CA, USA), called '054261On1M' (Design ID 066335) developed at the Research Core Unit Transcriptomics (RCUT) of Hannover Medical School (Hannover, Germany). The microarray design was created in Agilent's eArray portal using a 1 × 1 M design format for mRNA expression as template. All non-control probes of design ID 026652 have been printed five times within a region comprising a total of 181,560 Features (170 columns × 1068 rows). Four of such regions were placed within one 1 M region giving rise to four microarray fields per slide to be hybridized individually (Customer Specified Feature Layout). Control probes required for proper Feature Extraction software operation were determined and placed automatically by eArray using recommended default settings.

An amount of 250 ng of total RNA were used for synthesis of aminoallyl-UTP-modified (aaUTP) cRNA with the 'Quick Amp Labeling kit, no dye' (#5190-0447, Agilent Technologies) according to the manufacturer's recommendations, except that reaction volumes were quartered and contained NTP-mix was exchanged by NTP Set (ATP, CTP, GTP, UTP) and aminoallyl-UTP (Fermentas, Thermo Fisher Scientific; Waltham, MA, USA); order numbers R1091, R0481, respectively). Final NTP concentrations used for in-vitro transcription were 2.5 mM (ATP, CTP, GTP), 1.88 mM UTP, and 0.62 mM aaUTP. The labeling of aaUTP-cRNA was performed by use of Alexa Fluor 555 Reactive Dye (#A32756; LifeTechnologies) as described in the Amino Allyl MessageAmp™ II Kit Manual (#AM1753; Life Technologies, Carlsbad, CA, USA) except that reaction volumes were quartered.

cRNA fragmentation, hybridization and washing steps were carried-out as recommended in the 'One-Color Microarray-Based Gene Expression Analysis Protocol V5.7', except that 500 ng of each fluorescently labeled cRNA population was used for hybridization.

Slides were scanned on the Agilent Micro Array Scanner G2565CA (pixel resolution 3 µm, bit depth 20). Data extraction was performed with the 'Feature Extraction Software V10.7.3.1' using the extraction protocol file 'GE1_107_Sep09.xml'.

Measurements of on-chip replicates (quintuplicates) were averaged using the geometric mean of processed intensity values of the green channel, 'gProcessedSignal' (gPS) to retrieve one resulting value per unique non-control probe. Single Features were excluded from averaging, if they (i) were manually flagged, (ii) were identified as Outliers by the Feature Extraction Software, (iii) lay outside the interval of '1.42 × interquartile range' regarding the normalized gPS distribution of the respective on-chip replicate population, or, iv) showed a coefficient of variation of pixel intensities per Feature that exceeded 0.5.

Averaged gPS values were normalized by global linear scaling: all gPS values of one sample were multiplied by an array-specific scaling factor. This factor was calculated by dividing a 'reference 75th Percentile value' (set as 1500 for the whole series) by the 75th Percentile value of the particular Microarray to be normalized ('Array I' in the formula shown below). Accordingly, normalized gPS values for all samples (microarray data sets) were calculated by the following formula:

$$normalized\ gPS_{Array\ i} = gPS_{Array\ i}\ x\ (1500/75^{th}\ Percentile_{Array\ i}) \quad (1)$$

Finally, a lower intensity threshold (surrogate value) was defined based on intensity distribution of negative control features. This value was fixed at 10 normalized gPS units. All of those measurements that fell below this intensity cutoff were substituted by the respective surrogate value of 10.

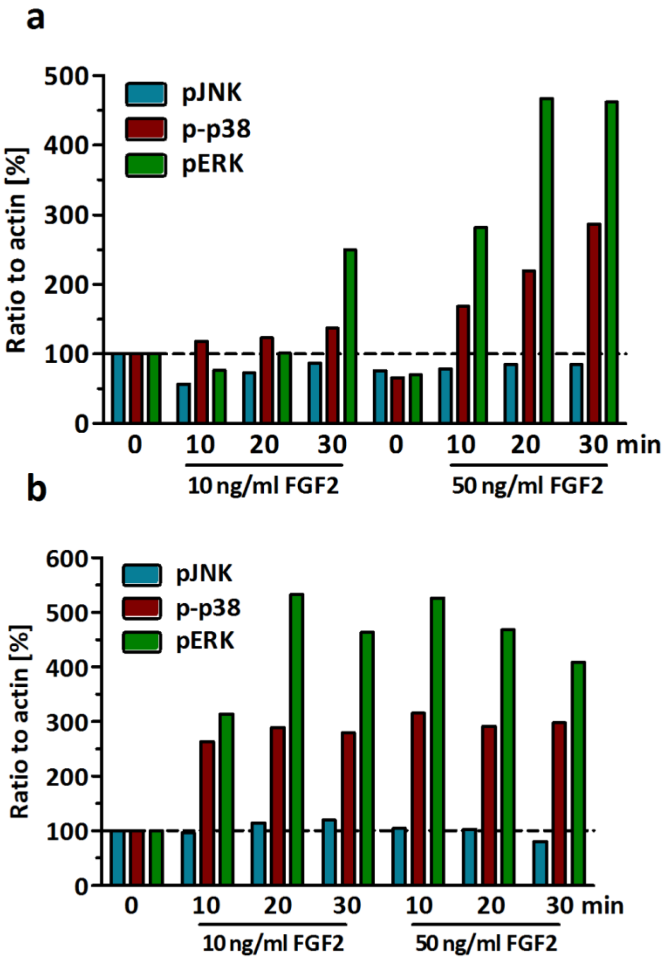

Supplementary Figure S1. Densiometric quantification of pERK, pJNK, and p-p38, normalized to beta actin and presented as [%] from two biological replicates(**a**,**b**).

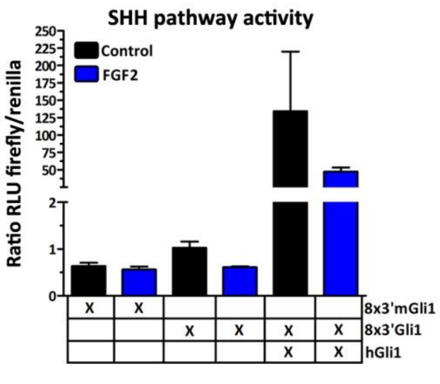

Supplementary Figure S2. Biological activity of secreted SHH in control media or FGF2-supplemented media in HEK293FT cells previously transfected with SHH luciferase reporter plasmids [16]. 8x3′mGli1 comprises mutated Gli1 binding sites, 8x3′Gli1 harbors eight repetitions of human Gli1 binding sites, and hGli1 encodes human *GLI1* as a positive control for the reporter assay.

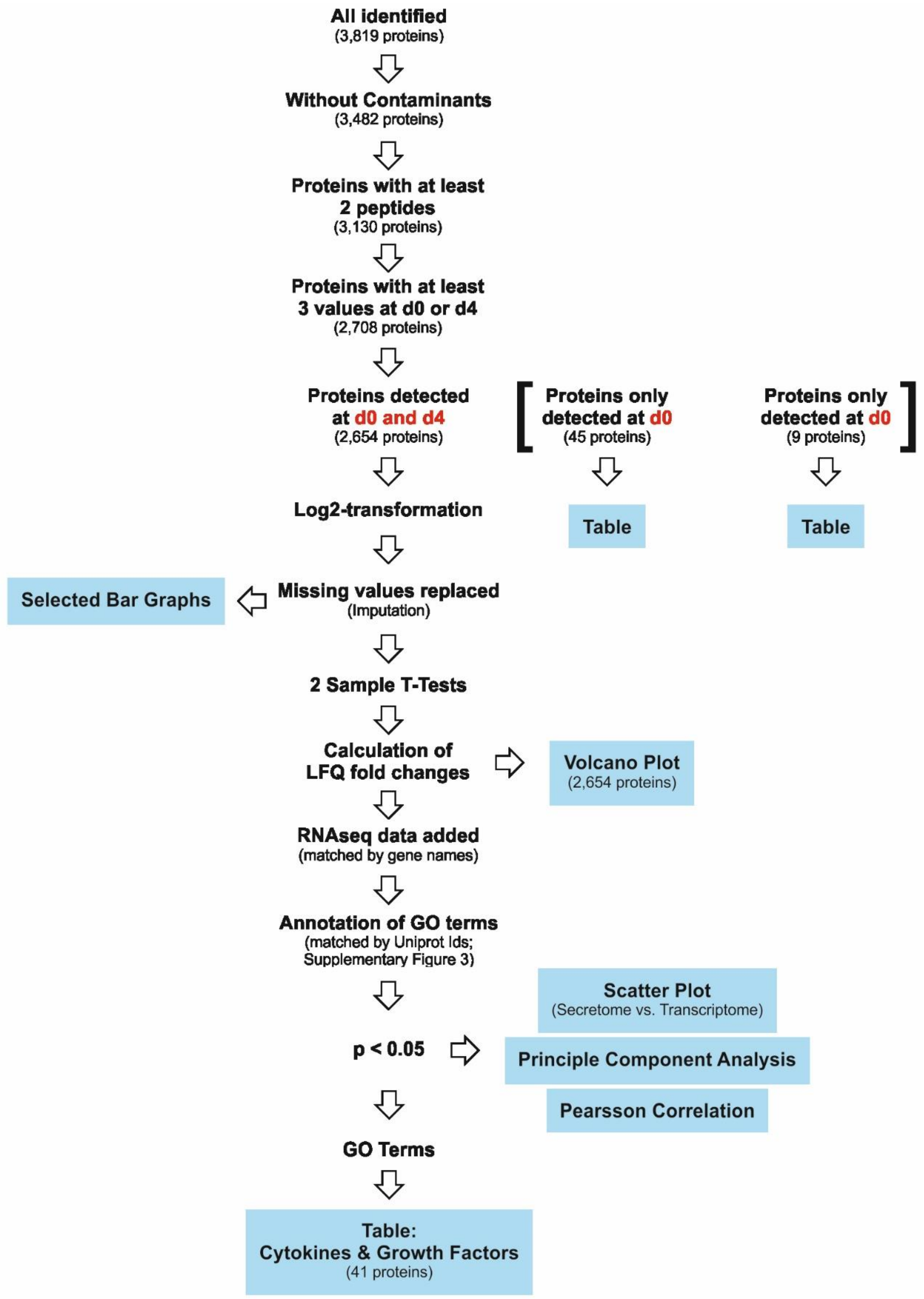

Supplementary Figure S3. Workflow of data processing using the Perseus software.

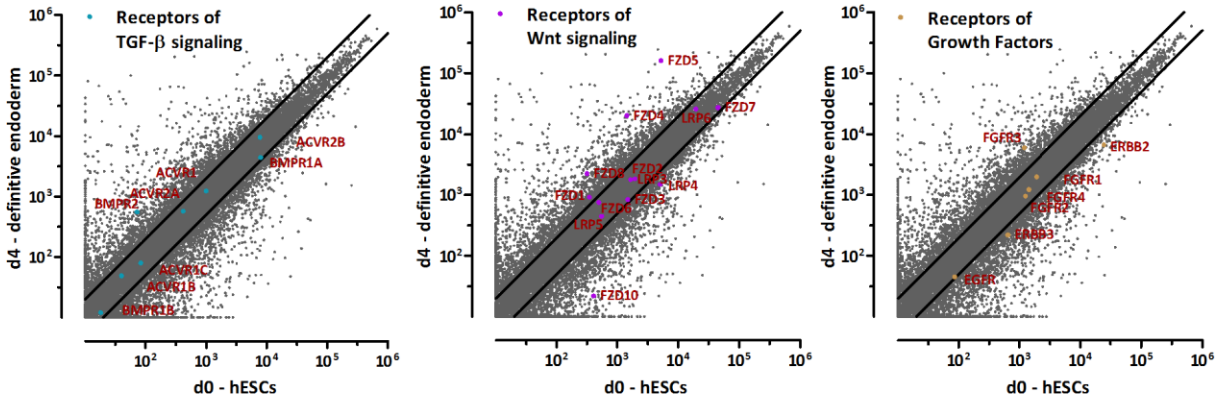

Supplementary Figure S4. Expression of receptors from the TGF-beta-, Wnt-, and FGF-pathway. The numbers on the axes represent the fluorescence values. Lower cut-off = 50.

Supplementary Table S1: Primer pairs for gene expression analysis.

Gene Symbol	Primer Sequence 5′-3′	Exon Spanning	Accession #
AFP	Fw: catgcttcagcagcttggtg<br>Rev: ctgcaatgacagcctcaagttg	Yes	NM_01134
CDX2	Applied Biosystems Taqman Assay<br>Hs01078080_m1	Yes	NM_001265.4
G6PD	Fw: aggccgtcaccaagaacattca<br>Rev: cgatgatgcggttccagcctat	Yes	NM_000402
HNF1B	Fw: gaggaatgcaacagggcagaatg<br>Rev: gaatgcctcctccttcctgcg	Yes	NM_000458
HNF6	Fw: cgctccgcttagcagcatgc<br>Rev: gtgtgttgcctctatccttcccatg	Yes	NM_004498
MNX1 (HLXB9)	Fw: tccaccgcgggcatgatc<br>Rev: gcttgggccgcgacaggta	Yes	NM_005515
PDX1	Fw: cgttccagctgcctttcccat<br>Rev: ccgtgagatgtacttgttgaatagga	Yes	NM_000209
SHH	Fw: tgtggccgagaagaccctag<br>Rev: caaagcgttcaacttgtcctta	Yes	NM_000193
SOX9	Fw: gcggaggaagtcggtgaagaacg<br>Rev: ctgggattgccccgagtgctc	Yes	NM_000346
TBP	Fw: caacagcctgccaccttacgctc<br>Rev: aggctgtggggtcagtccagtg	Yes	NM_003194
TBX1	Fw: gcagctagagatgaaggcg<br>Rev: aggtgggaaacatccgcctg	Yes	NM_080646
TUBA1A	Fw: ggcagtgtttgtagacttggaaccc<br>Rev: tgtgataagttgctcagggtggaag	Yes	NM_006009

Supplementary Table S2: Proteins exclusively detected at d0 and d4.

**Exclusively Detected at d0**		
**Gene Name**	**Protein Names**	**Mean LFQ (d0)**
SCG3	Secretogranin-3	1.13×10^{8}
MYO18B	Unconventional myosin-XVIIIb	3.42×10^{7}
DPPA4	Developmental pluripotency-associated protein 4	2.63×10^{7}
COL14A1	Collagen alpha-1(XIV) chain	1.52×10^{7}
BUB1B	Mitotic checkpoint serine/threonine-protein kinase BUB1 beta	1.26×10^{7}
CDH4	Cadherin-4	1.14×10^{7}
IDO1	Indoleamine 2,3-dioxygenase 1	1.04×10^{7}
ZNF483	Zinc finger protein 483	9.47×10^{6}
TFAM	Transcription factor A, mitochondrial	3.67×10^{6}
**Exclusively Detected at d4**		
**Gene Name**	**Protein Names**	**Mean LFQ (d4)**
SETD2	Histone-lysine N-methyltransferase SETD2	1.90×10^{8}
PIN1	Peptidyl-prolyl cis-trans isomerase NIMA-interacting 1	4.93×10^{7}
COL9A3	Collagen alpha-3(IX) chain	4.80×10^{7}
OTC	Ornithine carbamoyltransferase, mitochondrial	4.51×10^{7}
SEMA5A	Semaphorin-5A	4.21×10^{7}
CXCR4	C-X-C chemokine receptor type 4	3.04×10^{7}
GBA	Glucosylceramidase	2.52×10^{7}
ANGPT2	Angiopoietin-2	2.15×10^{7}
PCDH10	Protocadherin-10	2.06×10^{7}
ST3GAL2	CMP-N-acetylneuraminate-β-galactosamide-alpha-2,3-sialyltransferase 2	2.03×10^{7}
UBE4A	Ubiquitin conjugation factor E4 A	1.68×10^{7}
SRRM1	Serine/arginine repetitive matrix protein 1	1.58×10^{7}
PEG10	Retrotransposon-derived protein PEG10	1.58×10^{7}
TMOD1	Tropomodulin-1	1.54×10^{7}
SRPRB	Signal recognition particle receptor subunit beta	1.53×10^{7}
ICOSLG	ICOS ligand	1.53×10^{7}
ITGA4	Integrin alpha-4	1.49×10^{7}
COLEC12	Collectin-12	1.49×10^{7}
FAM20B	Glycosaminoglycan xylosylkinase	1.41×10^{7}
NUBP2	Cytosolic Fe-S cluster assembly factor NUBP2	1.38×10^{7}
TFPI2	Tissue factor pathway inhibitor 2	1.36×10^{7}
WNK1	Serine/threonine-protein kinase WNK1	1.35×10^{7}
TP53RK	TP53-regulating kinase	1.31×10^{7}
Uniprot ID Q9P1D1	N/A	1.19×10^{7}
CLSTN2	Calsyntenin-2	1.14×10^{7}
TFCP2	Alpha-globin transcription factor CP2	1.04×10^{7}
SNX9	Sorting nexin-9	1.02×10^{7}
TRMT1	tRNA (guanine(26)-N(2))-dimethyltransferase	1.00×10^{7}
OTUB2	Ubiquitin thioesterase OTUB2	9.83×10^{6}
GNL1	Guanine nucleotide-binding protein-like 1	9.27×10^{6}

PPP2R5A	Serine/threonine-protein phosphatase 2A 56 kDa regulatory subunit alpha isoform	8.71×10^6
ADAMTS12	A disintegrin and metalloproteinase with thrombospondin motifs 12	8.51×10^6
MPG	DNA-3-methyladenine glycosylase	7.15×10^6
GALNT18	Polypeptide N-acetylgalactosaminyltransferase 18	6.78×10^6
HBS1L	HBS1-like protein	6.45×10^6
INPPL1	Phosphatidylinositol 3,4,5-trisphosphate 5-phosphatase 2	5.58×10^6
PDLIM2	PDZ and LIM domain protein 2	5.47×10^6
UBE3A	Ubiquitin-protein ligase E3A	5.47×10^6
DPH5	Diphthine synthase	5.42×10^6
CSK	Tyrosine-protein kinase CSK	5.33×10^6
PIK3CB	Phosphatidylinositol 4,5-bisphosphate 3-kinase catalytic subunit beta isoform	4.93×10^6
SMCHD1	Structural maintenance of chromosomes flexible hinge domain-containing protein 1	4.60×10^6
Uniprot ID Q9Y250	N/A	4.56×10^6
PDE5A	cGMP-specific 3,5-cyclic phosphodiesterase	2.92×10^6
AK4	GTP:AMP phosphotransferase AK4, mitochondrial	2.69×10^6

Supplementary Table S3: GO-Term list.

GO Term	GO Number
Growth factor activity	[0008083]
Cytokine activity	[0005125]
Hormone activity	[0005179]
Epidermal growth factor receptor signaling pathway	[0007173]
Fibroblast growth factor receptor signaling pathway	[0008543]
Hepatocyte growth factor receptor signaling pathway	[0048012]
Nodal signaling pathway	[0038092]
Notch signaling pathway	[0007219]
Transforming growth factor beta receptor signaling pathway	[0007179]
Vascular endothelial growth factor signaling pathway	[0038084]
Wnt-signaling pathway	[0016055]
Retinoic acid receptor signaling pathway	[0048384]
Smoothened signaling pathway	[0007224]
